# Mediæval Remedies for the Plague

**Published:** 1898-06-18

**Authors:** 


### Modern Sociology.

### MEDIÆVAL REMEDIES FOR THE PLAGUE.

### I.—Prevention.

For over three centuries, from the appearance of the Black Death, in 1348, till the last record of the epidemic at Nottingham, in 1667, the "bubo plague" was practically endemic in England. During the latter part, at any rate, of this period a great revival of medical zeal was in progress. The classical authorities on medicine were diligently studied and translated, while a modern school, emancipated from musty rules of practice, followed hard on the footsteps of Paracelsus, and experimented with new remedies in daring emulation of their master. But the labour and ingenuity freely expended on other diseases stopped short of the plague. Owing, no doubt, to its cruel disregard of any distinction between doctor and patient, it was a complaint little in favour with the profession. Books of plague remedies are not wanting, but one is copied from another till the source is reached in some foreign authority who has probably himself copied his remarks from Jean de Vigo or the classics. Records of personal observation and practice on the part of the physician are extremely rare, and the same prejudices, the same ignorance, the same superstition encounters the reader in treatises written after the Great Plague, 1666, which are apparent in the first printed records of English medical practice, treasured in rare little Black Letter quartos.

That the physician was little in favour, and did not, on the whole, behave particularly well in times of dangerous epidemics, is clear from many contemporary allusions. It was a point eagerly debated whether they could justly be blamed for preferring safety by flight to gain, for the modern standard of devotion to duty and the advancement of science existed, if at all, among a few chosen spirits only.

One of the earliest books relating to the plague is "A Dialogue both Pleasant and Pitiful," 1573, by William Bullein, introducing a number of characters, and giving a vivid picture of the state of public feeling at the beginning of an outbreak when all are preparing to flee; and in this the physician certainly comes out very badly. He is painted opening his mind to Avarus, a patient like-minded with himself, and appears as a bitter atheist, masked with religion for the sake of his public, caring for nothing in the world but gain, and imposing on his credulous patients with tags of learning and superstitious observance. The practical object of the dialogue is to set forth remedies for the plague, but though a few ordinary prescriptions of herb drinks are appended, the greater part is given up to measures of prevention, and this it may be observed is the usual course in all plague manuals. In this branch of the subject the physician could practice with safety, his drugs and recommendations were eagerly sought after, and he could reap a golden harvest.

Like many other writers on the same topic, Bullein rehearses all the ordinary sanitary precautions which had been marked out by the deaths of successive generations as desirable. That crowded parts of the city should not be defiled with dungheaps, garbage or sewage; that the slaughter-houses should not cast forth their refuse into the open street; that the "shores" or open sewers running at the east part of the City should be occasionally purified; that water used for drink should not be polluted by infected matter, and that the carcases of cats, dogs, and horses should be removed from the waste parts of the town; such are some of the measures recommended most frequently, but to judge by the recurring orders, extending over more than a century, these simple reforms could never have been effectually carried out.

A common means for stopping infection was to burn fires in the streets to cleanse the air. One early writer recommends "the bells to be rung often and great ordnance discharged, thereby the air is purified." The same anonymous writer has strong views about burial, and urges the more enlightened precaution "that the corpses be laid a convenient depth in the ground, and not one coffin heaped upon another, and they so near the top of the earth as they now are." The force of this will be appreciated when it is remembered that Dr. Creighton attributes the extinction of plague in England to more sanitary methods of burial, in wooden coffins, after the Great Plague.

More personal recommendations with regard to prevention take the form of rigid dieting, of something medicated "to smell to" in the streets, of staying indoors, and of simple instructions to avoid fatigue and keep in good cheer. The directions about diet are curiously contradictory, no point being more oddly debated than the efficacy of onions and garlic. William Bullein believed its use to be fatal. He tells us "garlic, onions, leeks, rocket, radish are sold in every street in plague time," but emphatically warns his readers they "are means for to bring the same"; and he has many subsequent authorities to agree with him in absolutely forbidding their use. But the College of Physicians, in their manual, plagiarised from stock authors and issued by authority in 1636 and after, prescribe "garlic with butter and a clove or two," to be eaten first thing by all during an epidemic; others prescribe it minced small and boiled in milk, and no one seems to have perceived that the uncertainty arose from its having no effect whatever. Andrew Borde, writing in 1553, forbids during plague seasons the use of any kind of herb in the potage, which was the ordinary drink of the peasant, "thorow the infection of the air, the which doth corrupt the herbs." But the ordinary practitioner was not in accord with him, for we find many herb drinks confidently recommended as safe preventatives. Nothing can illustrate more forcibly the helplessness of the medical profession in face of this disease than the concoction prescribed by the College of Physicians to those of the "richer sort," brewed of hartshorne, pearle, corall, tormentill, citron pill, hyacinth stone, bezoar stone, east hunicorne's horn, &c. The "poorer sort" must have been better off with their "walnuts, figs, and rue," or "sorell and wormwood, drunk fasting." Sorell and rue were a very usual preventative, and appear in nearly all lists of simples for this purpose. Violets, spinach, borage, bugloss, scabious, gentian, and valerian were also very popular. "For delicate people, bread and cinnamon, or bread and butter, for butter is not only a preservative against the plague but against all other venom and poisons."

